# In Memory of Dr. Ratip Kazancıgil

**DOI:** 10.4274/balkanmedj.galenos.2019.2019.4.0003

**Published:** 2019-07-11

**Authors:** H. Murat TUĞRUL

**Affiliations:** 1 Retired Faculty of Department of Medical Microbiology, Trakya University Faculty of Medicine, Edirne, Turkey

Dr. Ratip Kazancıgil, MD, died on 12^th^ August 2017. He was a practioning physician, malaria combater, researcher, lecturer, manager, man of culture and volunteer work, a retired member of teaching staff of department of History of Medicine and Deontology of Faculty of Medicine of Trakya University in Edirne, Turkey (Figure 1).

We shall from now on remember him with his books, papers and scientific conferences, but he was mostly known as a modern day dervish for his humility, wisdom, relentless quest for new projects and with no concern for financial gains. He would strife till the end for efficient executions with follow ups for completion. All his works have been scientifically based, documented with cross references (1,2,3,4,5).

He started as a student of University Istanbul, Faculty of Medicine in 1937 and graduated in 1943. After completing his military service as conscript medical officer, he was posted to province of Aydın health directorate malaria combat department central unit as doctor to serve his compulsory medical service in 1946. For four years he treated malaria patients as well as led the efforts to dry out the mosquito breeding grounds of swamps in the area. As a result of his successful work, Dr. Kazancıgil was appointed as head of the malaria combat units in province of Edirne in 1950. He played a leading role in eradication of malaria in whole Thrace Region of Turkey. For his outstanding accomplishments he was received many awards from Ministry of Health of Turkey during this 11 years of service.

Dr. Kazancıgil was appointed as medical director of Edirne province in 1961. During his tenure of this post till his “first official” retirement in 1985 he started many important initiatives. For efficient execution of medical works in the rural areas he instigated training of nurses and midwifes, opening of health homes and centers and establishing broad based maternity health support services. Community based social health services were established at wide scale without direct contribution from the state. During this period, province of Edirne became exemplary lead province for social well fare organization. This achievement earned Dr. Kazancıgil the Prof. Nusret Fişek Public Health Award from Turkish Medical Council. His managerial achievements were also extended to as deputy Major of Edirne and Director of department of youth and sport affairs of Edirne province.

Dr. Kazancıgil obtained his PhD in History of Medicine and Deontology with his thesis under the supervision of Professor Süheyl Unver of University of Istanbul titled Health institutions in Edirne province of Ottoman Empire and their staffing in the years 1362 to 1920.

He was able to devote more time to scientific research work on becoming assistant professor and founding member of Medical History and Deontology department of Trakya University and worked as pioneering member for the development of this department. He worked as a member of teaching staff in the Faculty of Medicine till his “second” retirement and he was so much liked by his students that he was elected by them as best teaching staff of the year of the faculty twice.

He supervised 3 post licence thesis and set up a library, considered as workshop of the department and enhanced it with his personnel contribution of scientific and general knowledge books he has gathered over his working life.

He is author of 27 books and 127 papers mainly on general health and history of Edirne. He had a lifelong dream of establishment of a museum of health. After long and pain staking work he managed to have it built and completed as part of the Trakya University in the grounds of Sultan Beyazıt historical site. This museum was awarded the prize of European Museums Union in 2004.

His linguistic skills and through knowledge of ottoman Turkish language and its script was instrumental in his research into history of Edirne, its culture, health and welfare institutions. He has meticulously rewritten many source books in current Turkish in abridged form for the benefit of fellow researchers.

His papers were mostly on the subjects of health, history and personalities such a Professor Süheyl Ünver, Dr. Rıfat Osman Bey, Hafız Rakım Ertur, governor of Edirne Hacı İzzet Pasha. He has written four monographies on Professor Süheyl Ünver, Dr. Rıfat Osman Bey, Hafız Rakım Ertür, governer of Edirne Hacı İzzet Pasha.

He believed and considered medical doctors as flowers grown in green houses in need of constant care and be free of financial concerns.

For his lasting memory, the lecture halls building of the faculty of medicine of the university bears his name as a show of respect he has earned through years and value given to his achievements by his fellow staff members and the students (Figure 2).

## Figures and Tables

**Figure 1:**
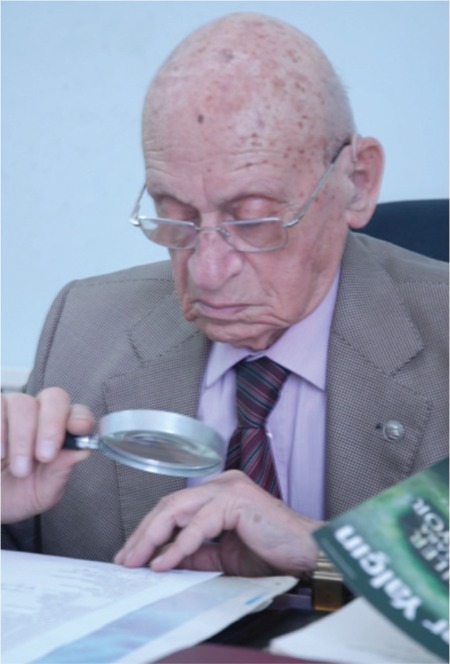
This picture of him exactly shows his post-retirement life.

**Figure 2:**
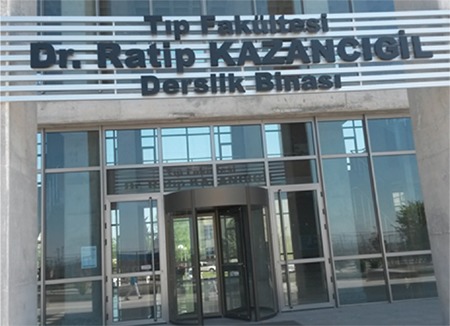
Lecture Halls building of faculty of medicine.

## References

[1] Tuğrul HM, Gökçe N (2010.). A Life Dedicated to Edirne and Public Health. Acar Publishing, İstanbul.

[2] Gökçe N, Tuğrul HM (2010.). On the 90th anniversary of his life a gift to Ratip Kazancıgil. Acar Publishing, İstanbul.

[3] Bilar E (2010.). Dr. Ratip Kazancıgil in the Cultural Life of Edirne. Çevik Publishing.

[4] Tuğrul HM Dr. Ratip Kazancıgil as an academician. Ottoman Health Traditions Meeting, 7-8 September 2018 Sultan II. Bayezid Külliyesi. Edirne..

[5] Kazancıgil R (2017). Being a medical doctor today trough the eyes of a ninety-five year old physician. Yeni Tıp Araştırmaları, İstanbul.

